# Longitudinal analysis of SARS-CoV-2 reinfection reveals distinct kinetics and emergence of cross-neutralizing antibodies to variants of concern

**DOI:** 10.3389/fmicb.2023.1148255

**Published:** 2023-03-29

**Authors:** Antonio Facciuolo, Jill Van Kessel, Andrea Kroeker, Mingmin Liao, Jocelyne M. Lew, Darryl Falzarano, Alyson A. Kelvin, Volker Gerdts, Scott Napper

**Affiliations:** ^1^Vaccine and Infectious Disease Organization (VIDO), University of Saskatchewan, Saskatoon, SK, Canada; ^2^Department of Veterinary Microbiology, University of Saskatchewan, Saskatoon, SK, Canada; ^3^Department of Biochemistry, Microbiology and Immunology, University of Saskatchewan, Saskatoon, SK, Canada

**Keywords:** COVID-19, duration of immunity, SARS-CoV-2 reinfection, cross-neutralizing antibodies, Delta (B.1.617.2), Omicron (BA.1)

## Abstract

The ongoing evolution of SARS-CoV-2 continues to raise new questions regarding the duration of immunity to reinfection with emerging variants. To address these knowledge gaps, controlled investigations in established animal models are needed to assess duration of immunity induced by each SARS-CoV-2 lineage and precisely evaluate the extent of cross-reactivity and cross-protection afforded. Using the Syrian hamster model, we specifically investigated duration of infection acquired immunity to SARS-CoV-2 ancestral Wuhan strain over 12 months. Plasma spike- and RBD-specific IgG titers against ancestral SARS-CoV-2 peaked at 4 months post-infection and showed a modest decline by 12 months. Similar kinetics were observed with plasma virus neutralizing antibody titers which peaked at 2 months post-infection and showed a modest decline by 12 months. Reinfection with ancestral SARS-CoV-2 at regular intervals demonstrated that prior infection provides long-lasting immunity as hamsters were protected against severe disease when rechallenged at 2, 4, 6, and 12 months after primary infection, and this coincided with the induction of high virus neutralizing antibody titers. Cross-neutralizing antibody titers against the B.1.617.2 variant (Delta) progressively waned in blood over 12 months, however, re-infection boosted these titers to levels equivalent to ancestral SARS-CoV-2. Conversely, cross-neutralizing antibodies to the BA.1 variant (Omicron) were virtually undetectable at all time-points after primary infection and were only detected following reinfection at 6 and 12 months. Collectively, these data demonstrate that infection with ancestral SARS-CoV-2 strains generates antibody responses that continue to evolve long after resolution of infection with distinct kinetics and emergence of cross-reactive and cross-neutralizing antibodies to Delta and Omicron variants and their specific spike antigens.

## Introduction

In late 2019, severe acute respiratory syndrome coronavirus 2 (SARS-CoV-2) was first characterized in Wuhan China (Zhu et al., 2020). In the absence of pre-existing immunity, this newly emergent pathogen spread rapidly around the globe reaching pandemic status just months after its detection. The COVID-19 pandemic, as caused by SARS-CoV-2, continues to have devastating economic and public health consequences. Given the increasing number of people who have recovered from SARS-CoV-2 infection, a central question for anticipating the impact on future infections by both existing and potentially emerging variants is understanding the duration of naturally acquired immunity. While reinfection with SARS-CoV-2 is to be anticipated (Abu-Raddad et al., 2021; Prado-Vivar et al., 2021; Tillett et al., 2021), the extended duration of time from initial infection and emergence of novel virus variants emphasizes the need to better understand the duration of immunity to SARS-CoV-2 reinfection and the extent of cross-protection against emerging variants. While several safe and effective vaccines have been developed, their implementation on a global scale remains a daunting challenge. Understanding the extent to which individuals can be reinfected will be critical for considerations of convalescent patients, cross-protection afforded against emerging variants of concern, as well as anticipating the trajectory of COVID-19.

As with other pathogenic human coronaviruses, the most established correlate of protection for SARS-CoV-2 is virus-specific neutralizing antibodies (Khoury et al., 2021). Albeit with distinct kinetics, naturally acquired immunity to severe acute respiratory syndrome coronavirus (SARS-CoV) and Middle East respiratory syndrome coronavirus (MERS-CoV) generates antigen-specific and virus neutralizing antibodies that can last more than 18 months (Zhu, 2004; Liu et al., 2006; Alshukairi et al., 2016; Payne et al., 2016; Mubarak et al., 2019; Memish et al., 2020). Due to the short duration of the SARS-CoV outbreak, and the lack of observational cohort studies to assess MERS-CoV reinfection, questions remain as to whether these long-lasting antibodies protect against reinfection (Memish et al., 2020; Al-Tawfiq et al., 2021). By contrast, for seasonal coronaviruses responsible for acute respiratory illness, a longitudinal, serological analysis of human CoV-specific antibodies showed that 12 months is the most frequently observed time-point for reinfection indicating that the naturally acquired immunity to these coronaviruses does not provide long-term protection to reinfection (Edridge et al., 2020). One of the earliest findings was that COVID-19 severity strongly correlated with greater levels of antigen-specific antibody responses (Hansen et al., 2021), however,it remains to be fully understood the extent in which this correlation translates to protection against reinfection and whether these stable antibody responses mediate long-term protection.

To define duration of the immunity to SARS-CoV-2, investigations have typically relied on quantification of either antigen-specific antibody responses or antibody-mediated virus neutralization, coupled with observational descriptive cohort studies, to determine reinfection rates. Following convalescence, SARS-CoV-2-specific IgG antibodies show a rapid decrease over the first 4 months, followed by a gradual decline with approximately 80%, or more, of infected individuals seropositive after one-year (Gluck et al., 2021; Turner et al., 2021; Vanshylla et al., 2021). In addition to waning of SARS-CoV-2-specific antibodies in blood, convalescence includes a prolonged period of memory B cell maturation with peak spike- and RBD-specific memory B cells responses at 6 months (Dan et al., 2021), a decline in SARS-CoV-2-specific CD4+ and CD8+ T cells within the first 6 months, and the generation of long-lived plasma cells (Turner et al., 2021). In the context of these immunological events during convalescence, a recent meta-analysis of observational descriptive cohort studies reported that protection from reinfection ranged from 69 to 98%–when only considering protection from symptomatic reinfection the pooled average was 92% (Petras, 2021). The effective period of protection was not significantly different up to 7 months following initial infection, however, in the aged population (> 65 years of age) protection was significantly reduced to 47% (Hansen et al., 2021). Retrospective cohort studies early in the pandemic found no evidence to indicate increased reinfection rates due to the B.1.1.7 variant in individuals with prior exposure to ancestral Wuhan strain (Hall et al., 2021; Lumley et al., 2021). In a recent retrospective cohort study, prior infection with ancestral Wuhan strain was effective in protecting against disease following reinfection with more antigenically distant variants of concern, such as Omicron (Chin et al., 2022).

While investigations of levels and durations of antigen-specific antibodies, virus neutralizing titers, and retrospective observational descriptive cohort studies all support the hypothesis of a window of protection against SARS-CoV-2 reinfection, the duration of that period has been defined by layering immunological data with observational cohort studies. Obtaining this information requires controlled investigation of reinfection that can only be evaluated within the context of animal models. To date, ferrets and hamsters are the primary animal models of SARS-CoV-2 infection. Of those, the Syrian hamster model has proven highly effective, in particular in demonstrating some of the human clinical manifestations of the disease in terms of weight loss and lung pathology (Imai et al., 2020; Sia et al., 2020).

Several reinfection studies in the hamster model evaluated protection within 20–30 days following primary infection. In both young and mature (>7 months) hamsters infection-acquired immunity afforded protection from disease, limited the recovery of infectious virus from the lungs, and reduced transmission to littermates (Imai et al., 2020; Brustolin et al., 2021; Nunez et al., 2021; Selvaraj et al., 2021). In all studies, infectious virus was recovered from tissues in the upper respiratory tract, but only for a short period following reinfection, indicating that infection-acquired immunity is superior at restricting viral replication in the lower than upper respiratory tract. Furthermore, SARS-CoV-2 ancestral strain Wuhan challenged hamsters were protected from reinfection with B.1.1.7 demonstrating that infection-acquired immunity affords cross-protection against this heterologous, albeit similar, variant of concern (Nunez et al., 2021). Of the few studies that have investigated long-term (>6 months) infection-acquired immunity in the hamster model it is evident that primary infection with strains similar to ancestral Wuhan can cross-protect against symptomatic reinfection with antigenically distinct variants of concerns like Delta and Omicron (Halfmann et al., 2022a,b; Stauft et al., 2022).

In the current study we employ a hamster model of SARS-CoV-2 infection to evaluate duration of immunity in hamsters by reinfecting at 2, 4, 6, and 12 months. Immunity to reinfection was evaluated through comparison of clinical indications of disease, quantification of infectious virus and viral RNA loads in the lungs and upper airway tissues and measuring antigen-specific antibodies and virus neutralizing titers at each reinfection time-point. Additionally, we evaluated the capacity of the antibodies circulating in blood, and those recalled after reinfection, to cross-neutralize the B.1.617.2 (Delta) and BA.1 (Omicron) variants as an indicator of infection-acquired immunity induced by ancestral Wuhan strain to provide cross-protection. The work presented herein shows that infection-acquired immunity to ancestral Wuhan strain affords long-term protection against symptomatic reinfection up to one year. These results also provide new insight into the serological reactivity of circulating antibodies and those recalled from the B cell pool, at 6- and 12 months reinfection, to cross-neutralize Delta and Omicron variants and potentially provide long-term cross-protective immunity.

## Materials and methods

### Animal use and ethics statement

All experiments were completed at the University of Saskatchewan following regulations established by the Canadian Council on Animal Care and approved by the University of Saskatchewan Animal Care Committee (Protocol #20200016).

### Virus

The challenge virus was ancestral Wuhan strain SARS-CoV-2/Canada/ON/VIDO-01/2020 (GISAID accession number EPI_ISL_425177). SARS-CoV-2 variants used in virus neutralizing assays were B.1.167.2 (Delta) and BA.1 (Omicron). Virus was cultured in Vero-76 (ATCC CRL-1587) cells in Dulbecco’s Modified Eagle Medium (DMEM; Millipore-Sigma Cat. # D5796), 2% fetal calf serum (SAFC Millipore-Sigma Cat. # 12306C), penicillin-streptomycin (100 U/mL and 100 μg/mL; Gibco^®^ Cat. # 15140122), and 2 μg/mL TPCK-trypsin (Thermo Fisher Scientific Cat. # 20233); herein referred to as vDMEM media. The viral stock of the second passage was used to challenge the hamsters and in subsequent assays.

### Hamster model of SARS-CoV-2 infection and duration of immunity study design

**FIGURE 1:**
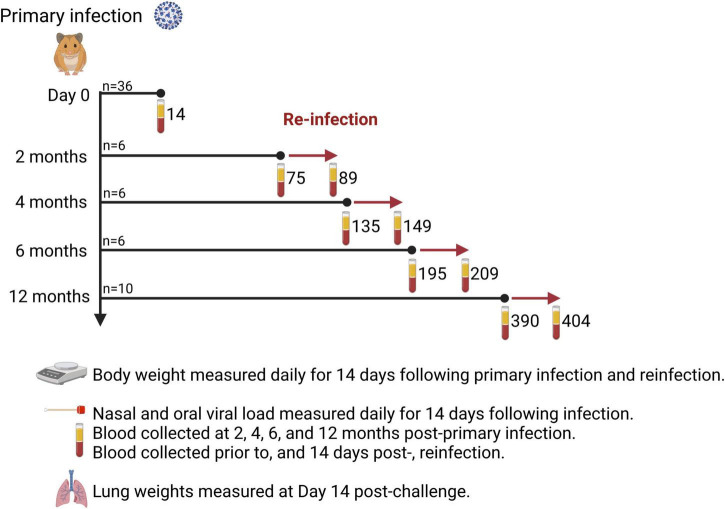
Study design. Thirty-six (36) Syrian hamsters were infected intranasally with 1 × 10^5^ TCID_50_ SARS-CoV-2 (ancestral Wuhan strain). Hamsters were randomly assigned to one of five cohorts to determine the time interval (2, 4, 6, or 12 months) for reinfection. Bodyweight was measured daily for 14 days after primary infection and reinfection. Hamsters were sacrificed 14 days after reinfection and lung weights recorded. Blood samples were taken prior to reinfection and 14 days after reinfection to measure spike S1- and RBD-specific IgG titers and virus neutralizing antibody titers to ancestral Wuhan strain, Delta, and Omicron. The number of days post-primary infection are indicated for each blood sampling time-point. Created with BioRender.com.

Male Syrian hamsters (*n* = 36) aged 6–7 weeks old were purchased from Charles River Laboratories. All animals were subjected to a primary intranasal challenge with SARS-CoV-2 virus by anesthetizing with isoflurane and delivering 50 μL per nare, *via* both nares for a total challenge dose of 1 × 10^5^ TCID_50_. Challenge and reinfection experiments, assays involving live virus, and housing of infected animals were carried out in the Vaccine and Infectious Disease Organization’s (VIDO) Containment Level 3 facility in Saskatoon, Canada. Following primary infection (*n* = 36), 4 animals died and were withdrawn from the study, therefore 32 animals participated in the cohort study. Four animals were randomly selected and euthanized to serve as the primary infection cohort representing 14 days post-infection (DPI). The remaining hamsters were randomly assigned to be reinfected at 2 mos (*n* = 6), 4 mos (*n* = 6), 6 mos (*n* = 6), and 12 mos (*n* = 10) post-primary infection (Figure 1).

A preliminary study was conducted with 36 male Syrian hamsters (6–7 weeks old, *n* = 12; 6 months old, *n* = 12; 12 months old, *n* = 12) to determine if there were age-dependent differences in clinical disease following primary SARS-CoV-2 infection. For each age-cohort, half of the hamsters (*n* = 6) were randomly assigned to receive a challenge dose of 1 × 10^5^ TCID_50_ and the other half (*n* = 6) served as uninfected controls. Uninfected hamsters aged 6–7 weeks old were used as age-matched controls when comparing lung weight ratios and bodyweights to young hamsters in the primary infection cohort. Six (6) month old, uninfected hamsters were used as age-matched controls when comparing lung weight ratios and bodyweight to the 2-, 4-, and 6 month reinfection cohorts. Twelve (12) month old uninfected hamsters served as age-matched controls when comparing lung weight ratios and bodyweight to the 12 month reinfection cohort.

### Sample collection schedule

Blood samples were collected in BD Vacutainer EDTA coated collection tubes for plasma separation before primary infection, prior to reinfection (2, 4, 6, and 12 months), and at 14 days post-infection (DPI) or 14 DP reinfection (DPR) to quantify antigen-specific IgG and virus neutralizing antibody responses. Daily clinical observation for general health conditions was conducted.

Nasal washes (0.3 mL) and oral swabs were collected immediately prior to primary infection, and at 1, 3, 5, 7, 9, 11, and 14 DPI, to quantify live virus and viral RNA. For RNA extraction, 140 μL of nasal wash or oral swab were added to buffer AVL containing carrier RNA as per manufacturer’s instructions and stored at −80°C (QIAamp Viral RNA isolation kit, Qiagen Cat#52906); the remaining volume was stored at −80°C and used for recovery of live virus. At necropsy, nasal turbinate, trachea, and lung tissues were collected for virological analysis. All tissues were stored in 1 mL RNAprotect (Qiagen Cat# 76106) at 4°C for up to one month. Tissues for recovery of live virus were stored at −80°C.

### Viral RNA extraction

The extraction of viral RNA from nasal washes or oral swab extracts was performed using Qiagen reagents (QIAamp Viral RNA Mini Kit Cat. # 52906). Briefly, 140 μL of nasal wash was added into 560 μL viral lysis buffer (Buffer AVL). The mixture was incubated at room temperature for 10 min. After brief centrifugation, the solution was transferred to a fresh tube containing 560 μL (equal volume) of 100% ethanol, and the tube was incubated at room temperature for 10 min. Viral RNA was then purified using QiaAmp Viral RNA Mini Kit and eluted with 60 μL of RNase-free water containing 0.04% sodium azide (elution buffer AVE).

Extraction of RNA from individual lung lobes (6 lobes) and nasal turbinate was done using approximately 100 mg of tissue. The tissues were homogenized in 600 μL of lysis buffer (RLT Qiagen) with stainless-steel beads in the TissueLyser II (Qiagen) for 6 min, at 30 Hz. The solution was then centrifuged for 5 min at 5,000 × *g*. A portion of supernatant equal to 30 mg of tissue was transferred to a fresh tube containing additional RLT buffer for a total volume of 600 μL. After 10 min, this was added to 600 μL of 70% ethanol, and the tube was incubated at room temperature for 10 min. Viral RNA was then purified using Qiagen RNeasy Mini Kit (Cat. # 74106) and eluted with 50 μL elution buffer.

### Real-time RT-qPCR

The RT-qPCR assays were performed on nasal washes, lung tissues, and nasal turbinate using SARS-CoV-2 specific primers for the E gene target. Qiagen QuantiFast RT-PCR Probe kits (Cat. # 204454) were used for RT-qPCR. Primer sequences were 5′-ACAGGTACGTTAATAGTTAATAGCGT-3′ and 5′-ATATTGCAGCAGTACGCACACA-3′, and 5′-ACACTA GCCATCCTTACTGCGCTTCG-3′ as the labeled probe. The RT-qPCR results were expressed in values of TCID_50_ equivalents (TCID_50_ Eq) per reaction. A standard curve of RNA detected by RT-qPCR was generated using serially diluted SARS-CoV-2 viral RNA. Briefly, viral RNA was extracted from viral stocks with known TCID_50_. Viral RNA samples were serially diluted (10-fold) to a final concentration range of 2 × 10^5^ to 2 × 10^1^ TCID_50_ per mL. The C_t_ values for individual samples were used with the standard curve to determine the TCID_50_ Eq/reaction. The RT-qPCR reactions were performed using the StepOnePlus™ Real-Time PCR System (Applied Biosystems™). Each 25 μL reaction consisted of 1X QuantiFast Probe RT-PCR Master Mix, 160 nM of primers, 80 nM of labeled probe, 0.25 μL QuantiFast RT Mix, and 5 μL of RNA. Reverse transcription was set for 10 min at 50°C followed by inactivation for 5 min at 95°C. Cycling conditions consisted of 40 cycles with denaturation for 10 s at 95°C and annealing/extension for 30 s at 60°C.

### Determination of viral titers

Tissues were homogenized in vDMEM using a Qiagen TissueLyser II. For nasal washes and oral swabs, 140 μL of saline solution was processed. A 1:10 dilution series of sample (starting at undiluted for tissues and at 1:10 for nasal and oral samples), in triplicate, was performed in vDMEM and added to Vero-76 (ATCC CRL-1587) cells in 96-well plates. Cells were incubated at 37°C for 1 h and replaced with fresh vDMEM. Cells were monitored daily for 5 days for cytopathic effect (CPE). On day 5, median tissue culture infectious dose (TCID_50_) was determined by microscopic evaluation of CPE. Tissue viral loads were calculated using the Spearman-Kärber method and data expressed as TCID_50_/mL or TCID_50_/g of tissue. Viral titration assays were performed only for samples in which viral RNA was detected by RT-qPCR.

### S1 and RBD-specific IgG antibody ELISA

Spike protein subunit S1 (S1) and Receptor-binding domain (RBD) proteins (ancestral Wuhan strain) were generated in-house by expressing in HEK293-T cells, concentrated and buffer exchanged by tangential flow, and purified by Nickel-sepharose agarose. Delta and Omicron S1 and RBD proteins were commercially sourced (Cedarlane Laboratories Cat. # 40591-V08H23, 40592-V08H90, 40591-V08H41, 40592-V05H3). Nunc MaxiSorp™ flat bottom 96-well microtiter plates (Invitrogen, Inc.) were coated by adding 100 μL/well of individual S1 (0.5 μg/mL) and RBD (0.5 μg/mL) proteins diluted in PBS, pH 7.4 and incubated overnight at 4°C. After washing with PBS supplemented with 0.05% v/v Tween-20 (PBS-T), wells were blocked with 5% w/v skim milk in PBS-T for 2 h at room temperature (RT). Four-fold serial dilutions of serum (starting at 1:100, in PBS-T) were added to duplicate wells and incubated at 37°C for 1 h. For detection of IgG antibodies, horseradish peroxidase conjugated, goat anti-hamster IgG (1:7,000 in diluent; Invitrogen™) was added to each well and incubated for 1 h at 37°C. After washing, each well was reacted with 50 μL o-phenylenediamine dihydrochloride (OPD; Thermo Fisher Scientific) diluted in Stable Peroxidase Substrate Buffer (Pierce™) for 30 min at RT in the dark. Reactions were stopped by adding 50 μL 2.5 M H_2_SO_4_ and absorbance measured at 490 nm using a SpectraMax Plus 384™ Reader (Molecular Devices). Antibody titers were determined using the reciprocal of the highest dilution that resulted in an absorbance value greater than the mean + 3 standard deviations (SD) of the absorbance value from control samples.

### Virus neutralization assay

Plasma samples were heat-inactivated for 30 min at 56°C. Serial Two-fold dilutions of plasma (starting at 1:20), in duplicates, were performed in vDMEM. A 1:1 mixture of virus solution (25 TCID_50_ in vDMEM) to diluted plasma sample (60 μL) was incubated for 1 h at 37°C, with 5% CO_2_ and then added to 90% confluent pre-seeded Vero-76 cells in 96-well flat-bottom plates. The plates were incubated at 37°C in a humidified chamber containing 5% CO_2_ for 5 days. The plates were observed under a light microscope on day 1 post-infection for contamination and on days 3 and 5 post-infection for CPE. The reciprocal of the lowest plasma dilution factor with no observed CPE at 5 days post-infection was defined as the end-point virus neutralization titer.

### Data and statistical analysis

Statistical analysis and data visualization was performed using GraphPad Prism 8.1 (GraphPad Software, Inc.). Assumptions of normal data distribution were performed for all datasets using the Shapiro-Wilk normality test. Differences were determined using a Mann Whitney test. For one-way ANOVA, Kruskal-Wallis tests were performed with *post-hoc* Dunn’s multiple comparisons test. *P*-values ≤ 0.05 were considered statistically significant.

## Results

### Study design and clinical observations following SARS-CoV-2 challenge

We conducted a pilot study with young and mature hamsters to identify if there were significant age-dependent differences in the clinical presentation of disease in this surrogate animal model. Hamsters aged 6–7 weeks (*n* = 12), 6 months (*n* = 12), and 12 months (*n* = 12) old were randomly assigned to one of two groups (*n* = 6/group). One group received an intranasal challenge with SARS-CoV-2 ancestral Wuhan strain and the other group serving as uninfected controls.

**FIGURE 2:**
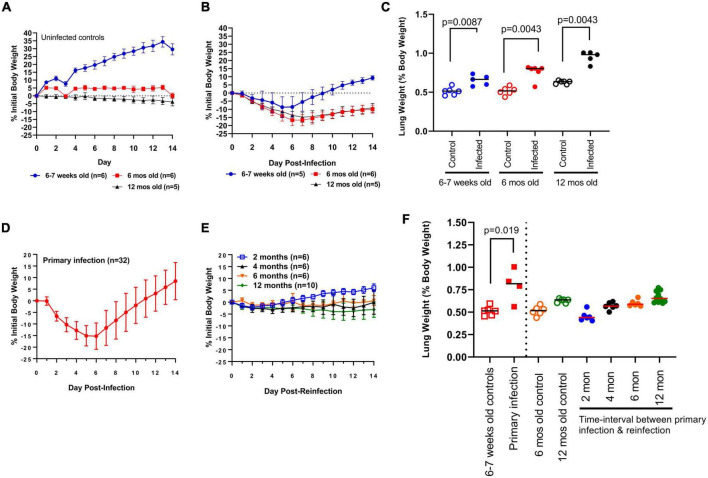
Clinical indications of disease. **(A)** Change in daily bodyweight for young (6–7 week-old) and mature (6- and 12 month-old) hamsters in the absence of infection. **(B)** Change in bodyweight in young (6–7 week-old) and mature (6- and 12 month-old) hamsters after primary infection with SARS-CoV-2 ancestral Wuhan strain. **(C)** Lung weight (as percent bodyweight) in young (6–7 week-old) and mature (6- and 12 month-old) hamsters after primary infection with SARS-CoV-2. Lung weights were measured at Day 14 post-infection. Each dot represents lung weight ratio for a single hamster, black horizontal bar represents group median. **(D)** Change in bodyweight in young (6–7 week-old) hamsters after primary infection and **(E)** reinfection at regular time-intervals with SARS-CoV-2. **(F)** Lung weight (as percent bodyweight) in young (6–7 week-old) and mature (6- and 12 month-old) hamsters in response to primary infection (solid squares) and reinfection (solid circles). Lung weights were measured at Day 14 post-challenge. Age-matched controls for young hamsters (open squares) and mature hamsters (6- and 12 month-old; open circles) are shown. Horizontal bars represent group median, each dot represents the lung weight ratio for a single hamster. Bodyweight data in panels **(A,B,D,E)** are expressed as changes to initial bodyweight prior to challenge, where dots represent the group mean and error bars represent one standard deviation. *P*-values were calculated using Mann Whitney test or Kruskal-Wallis one-way ANOVA.

Bodyweights for challenged and control hamsters were recorded daily (Figures 2A, B). Two hamsters (one in each of the 6–7 week-old and 12 month-old challenge cohort) reached predetermined humane intervention points of decreased body temperature and bodyweight loss. These hamsters were humanely euthanized and removed from the study analysis. As expected, young uninfected hamsters (*n* = 6) continued to increase in bodyweight over the 14 day observation window, whereas mature uninfected hamsters showed no significant change in bodyweight. When comparing the outcome of primary infection, young and mature hamsters showed mean peak body weight losses around similar time-points (days 5 and 6, and days 6 and 7, respectively) (Figure 2B). In each cohort, bodyweights began to increase following this peak period with a notable age-dependent exception. Young hamsters returned to their original body weight by day 9 and gained further weight up to day 14, whereas mature hamsters continued to show a mean 9.9% deficit in original bodyweight by the end of the 14 day observation window. Additionally, bodyweight loss was significantly greater (*p* < 0.05) in mature vs. young hamsters beginning at 6 DPI. In comparing lung weight, young and 6 month-old control hamsters had identical lung weight ratios (median 0.51, *n* = 5–6/group). By contrast, lung weight ratios were greater (median 0.63, *n* = 5) in 12 month-old control hamsters (Figure 2C). At 14 DPI greater (*p* < 0.05) lung weight ratios were observed in young (median 0.66, range 0.58–0.74), 6 month-old (median 0.80, range 0.57–0.81), and 12 month-old (median 0.98, range 0.83–1.00) hamsters relative to aged-matched controls (Figure 2C). Lung weight ratios were significantly greater in 12 month-old hamsters relative to young hamsters (*p* = 0.0027) and numerically higher relative to 6 month-old hamsters (*p* = 0.084) (Figure 2C). Consistent with previous reports, nuanced age-dependent differences exist in response to SARS-CoV-2 infection in the hamster model. Young hamsters were selected in this investigation to evaluate the long-term kinetics of antibody responses to SAR-CoV-2 in the context of infection-acquired immunity.

The primary infection consisted of 36 hamsters challenged intra-nasally with SARS-CoV-2 (Figure 1). Four hamsters succumbed to infection: two each at 7 and 8 DPI. Histological examination of the lung tissue of those 4 animals revealed severe active broncho-interstitial pneumonia. These hamsters also had prominent hemorrhage in their lung tissues that likely compromised lung function resulting in death (data not shown). These four animals were withdrawn from the study analysis. Of the surviving hamsters, four were euthanized to investigate clinical indications of disease, quantify viral RNA and infectious loads in the respiratory tract, and measure antibody responses. The remaining 28 hamsters were randomly assigned to one of four cohorts (Figure 1) and were reinfected with ancestral Wuhan strain at 2, 4, 6, and 12 months to evaluate the durability and kinetics of antigen-specific antibody responses to S1 and RBD antigens.

### Clinical indications of disease following reinfection

As an indicator of clinical disease, bodyweights were measured daily over 14 days and compared to the bodyweight prior to challenge (Figure 2D). The primary infection (*n* = 32) resulted in bodyweight loss starting at 2 DPI, peaking at 5 and 6 DPI (-15.1 ± 4.4 and −15.3 ± 5.7%, respectively; mean ± SD), a return to Day 0 weight at 12 DPI, and full resolution in all animals by 14 DPI. Hamsters reinfected at 2 months (*n* = 6) experienced mild bodyweight losses peaking at 2.1% at 2 DPI, whereas hamsters reinfected at 4 months (*n* = 6) experienced similar bodyweight losses (approximately 1.5 to 3%) that were sustained over the 14-day observation window (Figure 2E). Animals reinfected at 6 months (*n* = 6) experienced mild, but sustained, body weight loss (less than 2%), whereas hamsters reinfected at 12 months (*n* = 10) showed body weight losses ranging from 2–4% that were similarly sustained over 14 days post-reinfection (DPR) (Figure 2E). When comparing bodyweight changes in naïve 6- and 12 month-old hamsters (Figure 2B) to age-matched hamsters reinfected at 6 months and 12 months (Figure 2E) the modest bodyweight losses suggests infection-acquired immunity offers significant protection against disease up to 12 months after primary infection.

To further investigate clinical indications of disease, hamsters in each cohort were euthanized at 14 DPR and lung weights were measured and expressed as percent body weight (Figure 2F). Primary infection in hamsters resulted in the greatest lung weight ratio (0.80 ± 0.18; mean ± SD, *n* = 4) relative to aged-matched controls. Lung weight ratios in hamsters reinfected at 2 months (0.45 ± 0.06; mean ± SD, *n* = 6), 4 months (0.57 ± 0.04; *n* = 6), and 6 months (0.60 ± 0.04; *n* = 6) were not significantly different relative to 6 month-old control hamsters serving as age-matched controls (0.52 ± 0.05; *n* = 6) (Figure 2F). Similarly, lung weight ratios in hamsters reinfected at 12 months (0.67 ± 0.06; *n* = 10) were not greater relative to age-matched controls (0.63 ± 0.02; *n* = 5) (Figure 2F). Taken together, these clinical indications further support protection against severe disease up to 12 months following primary infection.

### SARS-CoV-2 in the upper respiratory tract

**FIGURE 3:**
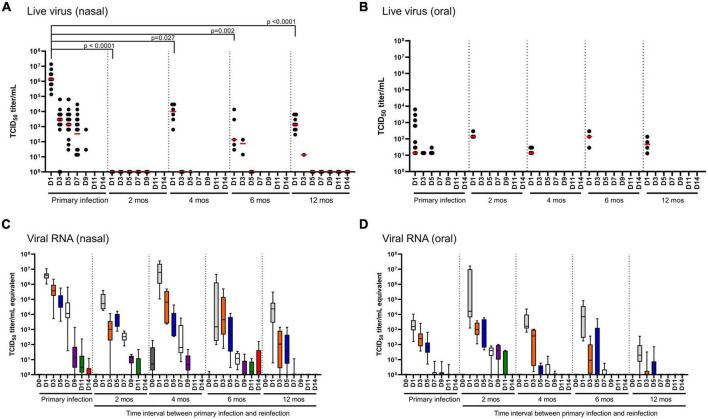
SARS-CoV-2 viral load in nasal and oral cavity. **(A,B)** Infectious virus and **(C,D)** total viral RNA in **(A,C)** nasal and **(B,D)** oral cavities monitored over 14 days in hamsters after primary infection (*n* = 32) and in the reinfection cohorts (2, 4, and 6 months, *n* = 6/time-point; 12 months, *n* = 10). In the scatter plots, each dot represents the amount of infectious virus recovered from a single hamster, the red horizontal line represents the group median. Each boxplot represents the median (interquartile range), error bars display min and max values. Each boxplot is uniquely color-coded based on the day of sampling. *P*-values were determined using a Kruskal-Wallis one-way ANOVA.

Infectious virus and SARS-CoV-2-specific RNA were quantified in nasal washes (Figures 3A, C) and oral swabs (Figures 3B, D) to determine viral shedding and persistence in the upper respiratory tract of hamsters following primary infection. In nasal washes, live virus was recovered from all hamsters following primary infection up to 7 DPI, and undetectable at 11 and 14 DPI (Figure 3A). Viral RNA in nasal washes revealed consistent findings with detection in all hamsters (*n* = 32) up to 7 DPI and marked decreases at 9, 11, and 14 DPI (Figure 3C). In the oral cavity, live virus and viral RNA was significantly lower than in the nasal washes (Figures 3B, D). Live virus was detected in a small proportion of the challenged hamsters and only up to 5 DPI. SARS-CoV-2 RNA was detected in the oral cavity for most animals up to 5 DPI, with marked decreases observed at 7–11 DPI, and undetectable at 14 DPI (Figure 3D).

Reinfection at 2 months resulted in no recovery of live virus from nasal washes (*n* = 6) and recovery of live virus from the oral cavity of three animals (*n* = 6) at 1 DPR (Figures 3A, B). In nasal washes, viral RNA was highest at 1, 3 5 and 7 DPR followed by a marked decrease at 9 DPR and undetectable in the most animals at 11 and 14 DPR (Figure 3C). In the oral cavity, viral RNA was highest at 1–5 DPR, marked decreases at 7 and 9 DPR, and undetectable in most animals at 11 and 14 DPR (Figure 3D).

Reinfection at 4, 6 and 12 months showed similar patterns of live virus recovery as was observed at 2 months (Figure 3). In the nasal washes, live virus was detected in most animals at 1 DPR and in 1–2 animals at 3 DPR in the 6- and 12 month reinfection cohorts (Figure 3A). When comparing live virus recovered in nasal washes at 1 DPR, we observed a significant (*p* < 0.05) decrease in viral burden (median 2–3 log_10_ lower) at all reinfection time-points when compared to primary infection. Thus, hamsters remain susceptible to reinfection in the upper respiratory tract, but the burden is significantly lower and clearance is much more rapid, occurring as early as 3 DPR. Viral RNA load in nasal washes at 6 and 12 months lend further support that infection-acquired immunity provides more rapid clearance as significantly (*p* < 0.05) less viral RNA is detected when compared to primary infection as early as D3 through D9. Similarly, in the oral cavity live virus is only detected at 1 DPR in 3–4 hamsters (*n* = 6–10/group), but there is no significant difference in median viral titers between that measured during primary infection or that measured at any of the reinfection time-points (Figure 3B); this is similarly reflected in the viral RNA loads (Figure 3D). In the oral cavity, viral RNA was detected in all animals (*n* = 6–10) at 1 DPR followed by a marked decrease at 3 and 5 DPR, and virtually undetectable by 7 DPR. Taken together, infection-acquired immunity provides more rapid clearance upon secondary exposure.

### SARS-CoV-2 in tissues and lower respiratory tract

**FIGURE 4:**
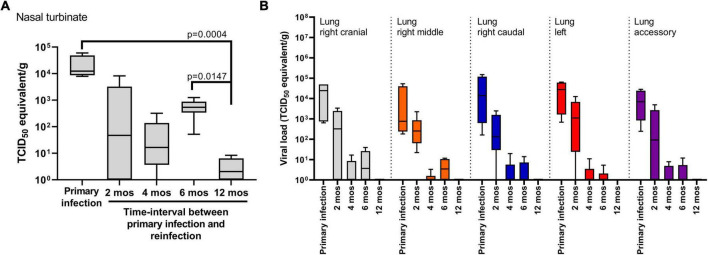
SARS-CoV-2 viral load in respiratory tissues. Viral RNA levels detected in **(A)** nasal turbinate and **(B)** lung tissues. Tissues were harvested at 14 days post-challenge in the primary infection cohort (*n* = 4) and in the cohorts reinfected at 2, 4, 6 (*n* = 6/time-point) and 12 months (*n* = 10). No live virus was recovered from tissue samples belonging to the primary infection cohort or the reinfection cohorts at 14 days post-challenge. Each boxplot represents the median (interquartile range), error bars display min and max values. *P*-values were determined using Kruskal-Wallis one-way ANOVA.

Nasal turbinate, trachea and lung tissue was collected at 14 DPI for the primary infection cohort and at 14 DPR for the reinfection cohorts to determine viral load by RT-qPCR and recovery of live virus (Figure 4). No live virus was recovered from any tissue samples collected at 14 DPI or 14 DPR in any of the cohorts. The four hamsters that succumbed to primary infection had detectable levels of infectious virus in nasal turbinate and lung tissues at the time of necropsy (Supplementary Figure 1). In trachea tissue, viral RNA was not detected at 14 DPI or 14 DPR except for one animal in the 4 month cohort (data not shown). By contrast, viral RNA was detected in the nasal turbinate of all animals (*n* = 4) at 14 DPI (Figure 4A). In the 2-, 4-, and 6 month reinfection cohorts (*n* = 6–10/group) there was a numerical, but not significant, decrease of viral RNA in the nasal turbinate relative to animals in the primary infection cohort. The lowest levels of viral RNA were observed in the 12 month reinfection cohort relative to the primary infection (*p* = 0.0004) cohort and the 6 month cohort (*p* = 0.014) (Figure 4A).

In lung tissue, five anatomical sites (right cranial, right middle, right caudal, left, and accessory) were sampled at 14 DPI for the primary infection cohort and at 14 DPR for the 2-, 4-, 6-, and 12 month reinfection cohorts (Figure 4B). Live virus was not recovered from any tissue samples for any of the animals. Viral RNA load was highest in lung tissue from the primary infection cohort and detected in all animals (*n* = 4). A 2 log_10_ range was observed between animals independent of the tissue site sampled, however, there were no significant differences in viral RNA load between tissue sites. Thus, to compare viral RNA burden between the primary and reinfection cohorts, we compared the average viral RNA load taken from all 5 anatomical sites across all animals in that cohort. The highest viral RNA loads were observed in the primary infection cohort, followed by a numerical, but not significant, decrease in the 2 month reinfection cohort (Figure 4B). Significant decreases in viral RNA load (*p* < 0.001) were observed in the 4-, 6-, and 12 month reinfection cohorts when compared to the primary and 2 month reinfection cohorts; no significant differences were detected between the 4-, 6-, and 12 month reinfection cohorts. Taken together, infection-acquired immunity provides more rapid clearance of the virus from the lungs upon reinfection with this observed effect lasting up to 12 months from the primary infection.

### Spike and RBD-specific antibody responses after primary infection

**FIGURE 5:**
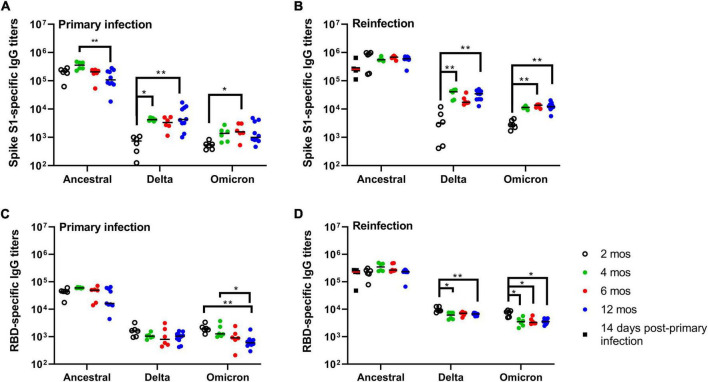
Durability and kinetics of SARS-CoV-2-specific antibody responses. **(A,B)** Spike (S1)- and **(C,D)** RBD-specific IgG antibody titers after **(A,C)** primary infection and **(B,D)** reinfection. **(A,C)** Antigen-specific IgG titers were measured at 2, 4, 6, and 12 months post-primary infection and **(B,D)** 14 days after reinfection at each of the time intervals. Antigen-specific IgG titers were measured against spike S1 subunit and RBD antigens derived from ancestral Wuhan strain SARS-CoV-2, Delta, and Omicron. Each dot represents an individual hamster, the black horizontal bar represents the group median. *P*-values were calculated using a Kruskal-Wallis one-way ANOVA. *p < 0.05; **p < 0.01.

Having established that primary infection of Syrian hamsters with SARS-CoV-2 ancestral strain Wuhan induces a durable and protective immune response up to 12 months, we next investigated the long-term kinetics of SARS-CoV-2-specific antibody responses in blood. IgG antibody responses in plasma were measured by ELISA using spike glycoprotein S1 subunit (S1)- and the receptor-binding domain (RBD) antigen derived from the ancestral Wuhan strain, and Delta and Omicron variants (Figure 5). Ancestral S1-specific IgG titers at 14 DPI were maintained over 12 months with a numerically higher median titer observed at 4 months, and significant (*p* = 0.0084) decrease at 12 months relative to titers measured at 4 months (Figure 5A). Ancestral RBD-specific IgG titers were maintained over 6 months with a modest (*p* = 0.0158) decline in median titers observed at 12 months relative to 14 DPI (Figure 5C). IgG antibody cross-reactivity to Delta and Omicron S1 and RBD antigen was significantly lower compared to ancestral antigen (Figures 5A, C). Cross-reactive IgG titers against Delta and Omicron S1 antigen were lowest at 2 months post-primary infection (300-fold and 374-fold lower, respectively) relative to ancestral S1 IgG titers (Figure 5A). Cross-reactive IgG antibodies against Delta and Omicron S1 antigen peaked at 4 months and were maintained to 12 months but remained significantly lower relative to those measured against ancestral S1 antigen (54 and 100 fold, respectively; Figure 5A). Cross-reactive RBD-specific IgG titers to Delta was over 20-fold less relative to ancestral RBD and were maintained out to 12 months (Figure 5C). Similar to Delta RBD-specific IgG titers, cross-reactive RBD-specific IgG titers to Omicron were 20-fold less relative to ancestral RBD however, these titers showed a modest but progressive decrease in median titers up to 12 months (Figure 5C). Taken together, S1- and RBD-specific IgG titers, as expected, were greatest toward ancestral antigen and significantly lower against Delta and Omicron antigen. The emergence of greater cross-reactive antibody titers around 4 months suggests an on-going evolution in the antibody response to SARS-CoV-2 long-after resolution of infection.

### Spike- and RBD-specific antibody responses after reinfection

Reinfection at 2, 4, 6, and 12 months resulted in a boost both in S1-specific and RBD-specific IgG titers when compared to IgG titers prior to reinfection (Figures 5B, D). Median S1- and RBD-specific IgG titers against ancestral antigen were not significantly different at any time-points suggesting SARS-CoV-2 antibody responses are maintained up to 12 months after primary infection. Consistent with our previous observations, cross-reactive S1-specific IgG titers against Delta and Omicron were lowest at 2 months with a marked increase observed at 4 months, and titers maintained out to 12 months (Figure 5B). Delta and Omicron S1-specific antibody titers were 156- and 225-fold, respectively, lower relative to ancestral S1 at 2 months, and approximately 18- and 46-fold lower at all other time-points. Cross-reactive RBD-specific IgG titers against Delta and Omicron were approximately equivalent across all time-points with statistically significant, but modest, decreases observed for the later time-points (Figure 5D). RBD-specific IgG titers were 49- and 86-fold lower for Delta and Omicron, respectively, relative to ancestral RBD. Taken together, reinfection boosts antigen-specific IgG antibody responses to ancestral S1 and RBD antigen with cross-reactivity evident against Delta and Omicron antigen.

### Virus neutralizing antibody responses in plasma following reinfection

**FIGURE 6:**
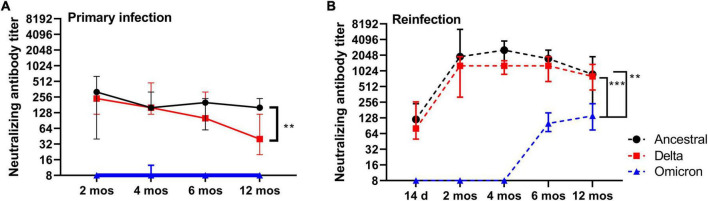
Virus neutralizing and cross-neutralizing antibody responses. **(A)** Anti-SARS-CoV-2 neutralizing antibody titers for ancestral Wuhan strain, Delta, and Omicron in plasma at 2, 4, 6, and 12 months post-primary infection. **(B)** Anti-SARS-CoV-2 neutralizing antibody titers for ancestral Wuhan strain, Delta, and Omicron in plasma after reinfection at 2, 4, 6, and 12 months post-primary infection. Each dot represents the group median (*n* = 6 for 2-, 4- and 6 month time-points; *n* = 10 for 12 month time-point), error bars represent 95% confidence interval. *P*-values were calculated using a Kruskal-Wallis one-way ANOVA. **p < 0.05; ***p < 0.0001.

To gain an understanding of the durability of functional antibody responses over time, we measured virus neutralizing (VN) antibody titers in plasma at regular intervals (2, 4, 6, and 12 months) in addition to after reinfection at 2, 4, 6, and 12 months against ancestral SARS-CoV-2. Additionally, we sought to determine if the S1- and RBD-specific IgG titers detected in plasma translate to functional antibody responses by performing virus neutralization assays with Delta and Omicron. Within the timeframe of this study Delta and Omicron (BA.1) were the predominant circulating strains. Median VN antibody titers against ancestral SARS-CoV-2 were lowest at 14 DPI (range 60–480; 70, median; *n* = 4), peaked at 2 months (range 40–640; 320, median; *n* = 6), and remained elevated at 4 months (range 160–320; 160, median; *n* = 6), 6 months and 12 months (Figure 6). Reinfection at 2, 4, 6, and 12 months significantly elevated plasma VN antibody titers relative to those measured prior to reinfection (Figure 6B). VN antibody titers recalled at 2 months (median 960, *n* = 6), 4 months (median 1280, *n* = 6), 6 months (median 1280, *n* = 6) were similar in magnitude, however, VN antibody titers measured at 12 months (median 480, *n* = 10) were significantly reduced relative to 4 months (*p* = 0.0008) and 6 months (*p* = 0.0176) (Figure 6). Taken together, VN antibodies are maintained in blood up to 12 months after primary infection, and reinfection significantly boosts VN antibody titers in plasma.

In contrast to median VN antibody titers against ancestral SARS-CoV-2 that were elevated over the course of 12 months, median VN antibody titers against the Delta variant showed a downward trend over the same time-points with the lowest titers observed at 12 months (*p* = 0.009) when compared to 2 months (Figure 6A). Reinfection, however, boosted median VN titers against Delta to levels equivalent to that against ancestral SARS-CoV-2 (Figure 6B). Cross-neutralizing VN antibody titers against Omicron were not detected at any time-point following primary infection, with exception of one hamster at 4 months (Figure 6A). However, VN cross-neutralizing antibodies against Omicron were detected at 6 and 12 months only after reinfection (Figure 6B). Omicron VN antibody titers were significantly (*p* < 0.05) lower relative to ancestral SARS-CoV-2 or Delta. These data suggest that circulating long-lived neutralizing antibodies induced by ancestral SARS-CoV-2 are maintained up to 12 months, but those that have the capacity to cross-neutralize Delta wane over time. Furthermore, the different rates of decline in neutralizing antibody titers against ancestral and Delta SARS-CoV-2 in combination with the emergence of cross-neutralizing antibodies against Omicron point toward distinct changes, or evolution, in the B cell and circulating antibody repertoire over time.

## Discussion

Understanding the extent to which individuals who recover from COVID-19 are susceptible to reinfection will be important considerations of individual responses, framing expectations of the potential to achieve and maintain herd protection through a combination of vaccination and naturally acquired immunity. The Syrian hamster provides a model that captures many aspects of COVID-19 in humans and affords the opportunity to carry out controlled experimental studies to investigate fundamental questions surrounding SARS-CoV-2 immunity. The current study provides the first one-year longitudinal analysis to chart out the long-term kinetics of antibody reactivity to the ancestral Wuhan strain and the capacity of these antibodies to cross-react and cross-neutralize antigenically distinct variants such as Delta and Omicron. This controlled investigation using an animal model revealed the maintenance and durability of antigen-specific and virus neutralizing antibody responses, the protection afforded to re-exposure at regular time-intervals over this period, and the potential of infection acquired immunity to ancestral SARS-CoV-2 to provide serological cross-immunity to variants of concern, such as Delta (B.1.617.2) and Omicron (BA.1).

Upon primary SARS-CoV-2 infection, both young (6–7 week-old) and mature (6- and 12 month old) hamsters show a significant reduction in bodyweight with mean weight loss of 15% of initial bodyweight, consistent with previous reports (Imai et al., 2020; Osterrieder et al., 2020; Rosenke et al., 2020; Sia et al., 2020). Consistent with our findings, previous studies have shown that infection acquired immunity in hamsters protects from clinical indications of disease such as weight loss and lung pathology following reinfection with ancestral SARS-CoV-2 up to 6 months after primary infection in young hamsters (Imai et al., 2020; Brustolin et al., 2021; Selvaraj et al., 2021; Field et al., 2022; Hansen et al., 2022) and longer protection of 9 months when the study started with aged hamsters (Nunez et al., 2021). We provide the first longitudinal analysis up to one-year in hamsters that demonstrates durable immunity to ancestral SARS-CoV-2 reinfection up to 12 months. In the current study, live virus was recovered from the upper respiratory tract of most animals up to 7 DPI after primary infection, and consistently recovered at 1 DPR at all time-intervals of reinfection except for 2 months (Figure 3). By contrast, viral RNA in the upper respiratory tract (nasal washes and oral swabs) was detected in most animals up to 5 DPI but depreciating viral RNA levels were observed at each subsequent time-interval for 7 to 14 DPR (Figure 3). Consistent with previous reports, infection-acquired immunity does not provide sterilizing immunity in upper respiratory tract, however, the reduction in viral RNA and infectious virus suggests it provides more rapid onset of clearance when compared to the primary infection, and this protective response in the upper respiratory tract lasts for 12 months. Although not addressed in the current study, convalescent ferret (Kim et al., 2021) and hamsters (Selvaraj et al., 2021) have reduced transmissibility to naïve littermates presumably as a function of lower viral loads and days of reduced shedding. Similarly, in humans, seropositive health care workers had lower viral loads in nasal and oropharyngeal swabs upon re-exposure when compared to their seronegative colleagues following primary exposure (Lumley et al., 2021).

In young hamsters, complete resolution of infection and disease occurs around 14 DPI (Osterrieder et al., 2020; Sia et al., 2020). We observed hamsters up to this time point after each reinfection to address protection from disease using clinical indications such as change in body weight and lung weight ratios. In previous studies, days 2 through 7 were used as endpoints to capture outputs such as live virus given that peak levels of viral RNA and sub-genomic RNA occur at 2 DPI (Brustolin et al., 2021). However, even at these earlier time-points, live virus was not recovered from the lungs of reinfected animals (Brustolin et al., 2021) or at later days (10 days) except for recovery from upper airway tissues (Imai et al., 2020). Therefore, similar to the protection afforded in the upper respiratory tract, infection acquired immunity also provides more rapid clearance of live virus from the lower respiratory tract.

With each emerging variant new questions are raised regarding the extent of cross-protection afforded by infection acquired immunity induced by a prior, antigenically distinct, SARS-CoV-2 strain. In previous studies, SARS-CoV-2 ancestral Wuhan strain infection in aged hamsters (>10 months) offered long-term (>6 months) heterologous protection to Alpha and Delta variants (Nunez et al., 2021; Stauft et al., 2022). In young hamsters, it is evident that primary infection with ancestral Wuhan strain can confer long-term protection against disease from both homologous and heterologous (Alpha, Beta, Delta, Omicron) reinfection (Field et al., 2022; Halfmann et al., 2022b; Hansen et al., 2022). Our study corroborates these findings by uniquely demonstrating homologous protection up to 12 months after primary infection. These findings in the Syrian hamster model mirror observations in retrospective cohort studies in humans showing that B.1.1.7 did not affect reinfection rates in those previously infected with ancestral Wuhan strain (Hall et al., 2021; Lumley et al., 2021). Considering the more recent variants, retrospective case studies have demonstrated that prior SARS-CoV-2 infection in humans (occurring before the predominance of the Delta wave) confers protection to Omicron (Chin et al., 2022). Given the larger genetic and phenotypic divergence of existing variants of concern, such as Delta and Omicron, fundamental questions regarding the capacity of ancestral Wuhan strain infection acquired antibodies to bind and cross-neutralize heterologous strains becomes critical. To best evaluate this in the context of our study we measured the capacity of infection acquired antibodies circulating in plasma and those recalled during reinfection with the ancestral Wuhan strain to cross-react and neutralize the Delta and Omicron (BA.1) variants. In previous reports, cross-reactive antibody responses were demonstrated against Alpha, Beta, and Omicron spike and RBD antigens following ancestral Wuhan strain infection (Field et al., 2022; Hansen et al., 2022). Our study provides a complete longitudinal analysis of antibody reactivity to spike and RBD antigens derived from ancestral Wuhan strain, Delta, and Omicron beginning at 2 months and at regular intervals up to 12 months post-infection. Despite the observation that S1- and RBD-specific IgG titers were maintained in plasma over one-year (Figure 5), cross-neutralizing antibody titers against Delta showed a progressive decline between 2 and 12 months (Figure 6) whereas neutralizing antibody titers against ancestral Wuhan strain showed no significant change; Omicron cross-neutralizing antibody titers were virtually undetectable at any time-point following primary infection (Figure 6). In humans, it has similarly been observed that neutralizing antibodies to ancestral Wuhan strain are maintained over months, and up to one year, but effectiveness against heterologous strains (i.e., variants) appears to wane (Edara et al., 2021; Haveri et al., 2021; Planas et al., 2021). Unique to our findings is that reinfection with ancestral Wuhan strain at all time-points up to 12 months significantly elevated neutralizing antibody responses in plasma with titers against ancestral Wuhan strain and Delta being virtually equivalent. Unique to this study is that cross-neutralizing antibodies to Omicron uniquely emerged at 6 and 12 months and were only detectable following reinfection (Figure 6). There are a few critical implications raised by these findings. First, quantifying levels of neutralizing antibodies in blood as a surrogate measure of protection (prior to a reinfection event) might not reflect the full potential of the host response to neutralize, or cross-neutralize, antigenically distinct variants. Further, measuring virus neutralization is necessary to evaluate the functionality of cross-reactive antibody responses detected. Second, given the observation of low VN antibody titers against Delta and Omicron prior to reinfection followed by high titers induced by reinfection suggests either a robust recall response of these cross-reactive antibodies from the memory B cell pool or can suggest that the repertoire of long-lived antibodies in blood may be distinct from that of the memory B cell pool. This finding merits the use of high-resolution analyses to resolve this question. Further, these data beg the question whether waning reactivity of these long-lived antibodies in blood to variants could impair rapid serological immunity or whether expansion of memory B cells occurs rapidly enough to compensate for this apparent waning. Collectively, these data suggest that naturally acquired immunity induced by ancestral Wuhan strain can generate cross-neutralizing antibody responses to emerging variant of concern that are antigenically distinct.

A unique perspective provided by this study is that reinfection boosts cross-reactive, functional antibodies to Delta to similar levels observed with the ancestral Wuhan strain and reveals functional antibodies to Omicron can evolve long-after resolution of infection. Although a limitation of this study was that we did not reinfect with heterologous strains, it is evident from previous studies in hamsters and humans that B.1 lineage can afford protection against disease to antigenically distinct variant such as Delta and Omicron (Chin et al., 2022; Halfmann et al., 2022a,b). Moreover, the emergence of unique antibody responses to these variants is consistent with immune profiling of memory B cells in hamsters at 4 months after primary infection (Horiuchi et al., 2021) and in humans up to 6 months post-infection (Sakharkar et al., 2021; Sokal et al., 2021). Our study warrants a similar analysis be performed at later time-points as much more divergent antibody responses begin to emerge around 6 months post-primary infection. These analyses have major implications for infection-acquired immunity as primary infection with Omicron displayed weaker cross-neutralization to ancestral Wuhan strain, Alpha, Beta and Delta than that reported when the primary infection was with ancestral Wuhan strain (Mohandas et al., 2022). Taken together, these studies suggest that the primary infection may be a critical determinant in the development of cross-neutralizing antibodies and warrants a similar longitudinal analysis using Delta and Omicron as the primary infection to determine the extent to which these viral variants display distinct kinetics and evolution in the B cell response relative to ancestral Wuhan strain. This could have major implications for vaccine antigen design and selection as the variant antigen sequence may inherently possess distinct properties than the ancestral antigen that could affect its retention in germinal centers and subsequently the continued maturation and diversification of B cell responses.

In summary, this study provides a more complete picture of the kinetics and evolution of SARS-CoV-2 immunity in the Syrian hamster model by evaluating immune responses out to 12 months post-primary infection and demonstrates that reinfection provides an added dimension in interrogating the expanded antibody repertoire. Given enough time between primary infection and secondary exposure B cell responses can evolve to generate cross-neutralizing antibodies against emerging variants. The extent to which these B cell responses continue to evolve, their durability beyond one-year, and the capacity of these cross-neutralizing responses to protect against symptomatic reinfection warrants further investigation.

## Data availability statement

The original contributions presented in this study are included in the article/Supplementary material, further inquiries can be directed to the corresponding author.

## Ethics statement

This animal study was reviewed and approved by the University Animal Care Committee (Protocol #20200016), University of Saskatchewan.

## Author contributions

ML and SN: conceptualization. SN: methodology. JV, JL, and AK: investigation. AF and SN: writing—original draft. AF, SN, AAK, DF, JL, JV, and VG: writing—review and editing. AF, AAK, and SN: formal analysis. AF: visualization. DF and VG: funding acquisition and resources. DF and SN: supervision. All authors contributed to the article and approved the submitted version.
