# Supplementary figures and images for: Longitudinal analysis of SARS-CoV-2 reinfection reveals distinct kinetics and emergence of cross-neutralizing antibodies to variants of concern
Source: Front Microbiol. 2023 Mar 29;14:1148255. doi: 10.3389/fmicb.2023.1148255 (PMC10090301; doi:10.3389/fmicb.2023.1148255)

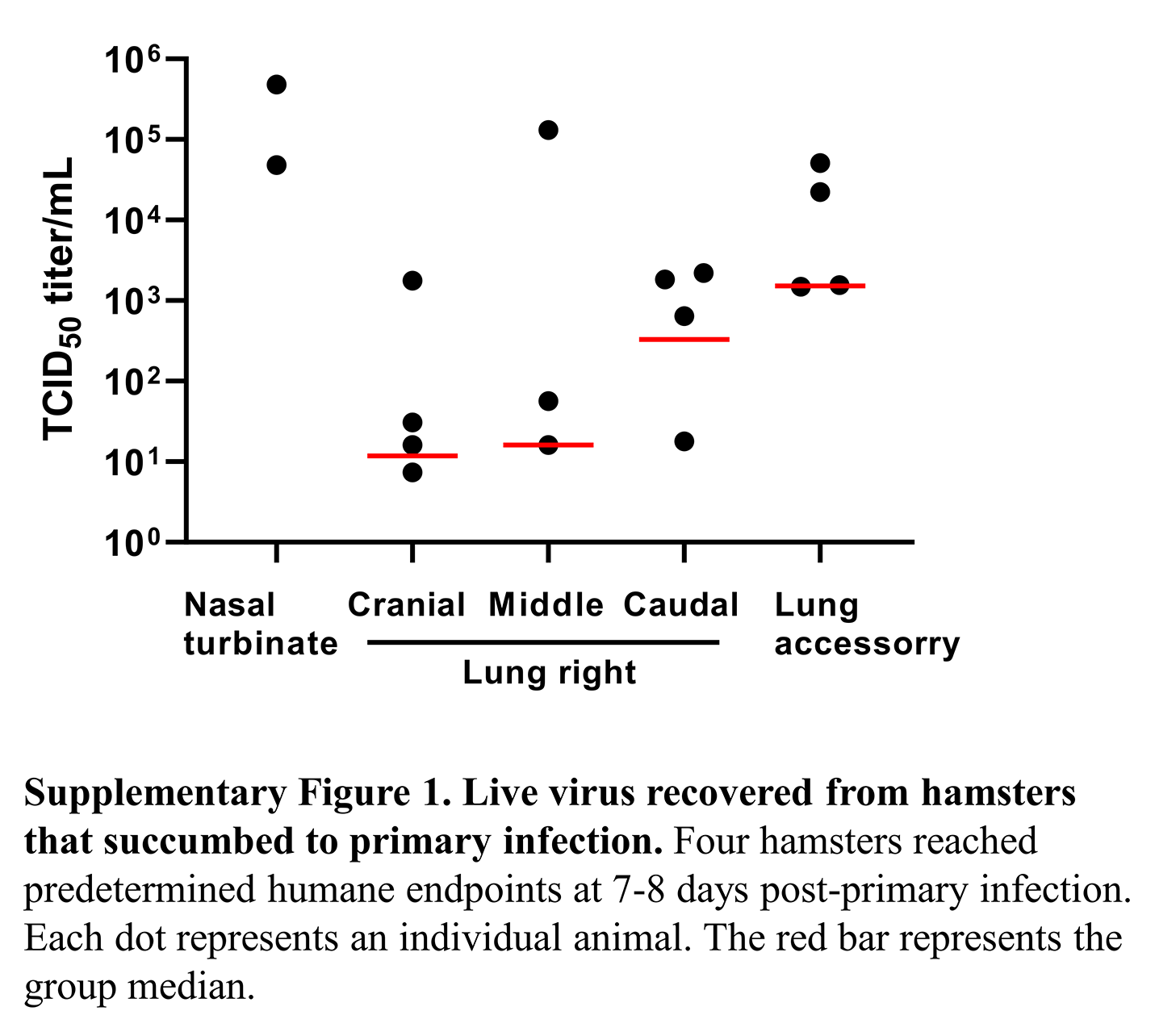

Supplement: Supplementary file 1 [file Image_1.TIF]
